# The use of a plate for fixation of the acetabulum

**DOI:** 10.25122/jml-2023-0310

**Published:** 2024-02

**Authors:** Nurgeldi Manap, Nagmet Mursalov

**Affiliations:** 1 National Scientific Center of Traumatology and Orthopedics named after Academician N.D. Batpenov, Department of Traumatology, Astana, Kazakhstan

**Keywords:** acetabular fracture, Stoppa approach, osteosynthesis, plate

## Abstract

This study aimed to share our experience of a self-developed plate for acetabular fracture fixation through the presentation of clinical cases. Eight patients with complex acetabular fractures (Letournel classification) underwent surgery using a modified Stoppa approach and the novel plate design between 2021 and 2023 at the National Scientific Center for Traumatology and Orthopedics. Criteria such as the mechanism of injury, type of fracture, surgical approach, intraoperative and postoperative complications, quality of reduction, and functional and radiological results were evaluated. All patients included in the study presented complex types of acetabular fractures according to the Letournel classification. In all cases, surgical interventions were performed using a modified Stoppa approach. The assessment of reduction quality was conducted based on the radiological standards established by Matta. The reduction in quality was excellent in two patients, good in four, and satisfactory in two. One patient developed a post-traumatic false joint requiring additional surgery. The remaining patients achieved fracture healing with satisfactory Harris Hip Score (HHS) scores, indicating good overall function. The results of the self-developed plate for acetabular fracture fixation in our series were satisfactory.

## INTRODUCTION

Acetabular fractures, although uncommon, occurring at a rate of three new cases per 100,000 inhabitants, pose a significant challenge for orthopedic surgeons [1,2]. These complex injuries typically result from high-energy trauma like motor vehicle accidents, pedestrian accidents, sports injuries, and falls from heights [1]. Open reduction and internal fixation (ORIF) continues to be the preferred method for managing dislocated acetabular fractures [3,4].

The treatment of acetabular fractures has undergone radical changes over the past 60 years. Prior to the 1960s, most acetabular fractures were managed conservatively. However, in 1964, R. Judet and E. Letournel first described the principles of acetabular surgery, which revolutionized the management of this injury [5]. Presently, a consensus among most authors is that open reduction and internal fixation of acetabular fractures should aim to provide early mobilization, rapid pain relief, and restore the anatomy of the hip to prevent the development of post-traumatic osteoarthritis [6,7].

The complex anatomical structure and severity of the injury necessitate a continuous search for and development of new surgical methods for managing acetabular fractures. In recent times, there has been extensive research on new approaches and devices for internal fixation in treating fractures involving the acetabulum. To enhance stability in fixation and clinical outcomes, we introduced a novel approach for treating fractures involving the acetabulum, using a specialized plate for acetabular fixation (Patent No. 7622 Republic of Kazakhstan). Our objective was to create a plate design that ensures better anatomical contouring, considering the specific nature of acetabular fractures while enhancing the quality of fixation, providing additional versatility, and expanding the range of metal constructs available for acetabular fracture surgery.

## MATERIAL AND METHODS

Eight patients with various acetabular fractures underwent surgery between 2021 and 2023 at the Department of Traumatology of the National Scientific Center of Traumatology and Orthopedics named after Academician Batpenov N.D. All surgeries utilized an anterior intrapelvic approach with a self-developed plate for acetabular fixation. This study included adult patients (aged 18 years or older) with acetabular fractures classified according to the Judet-Letournel system. All patients underwent surgery using the novel acetabular fixation plate and a modified Stoppa approach. We excluded patients with fractures involving the posterior wall of the acetabulum, those younger than 18, and those treated with other implant designs. Evaluation criteria included the mechanism of injury, fracture characteristics, surgical approach, intraoperative and postoperative complications, quality of reduction, functional outcomes, and radiological results. This research is registered on ClinicalTrials.gov by ClinicalTrials.gov ID: NCT06005753.

As part of the preoperative preparation, pelvic bone radiographs, computerized tomography (CT) with angiography, and three-dimensional (3D) rendering were performed. The day before surgery, a repeat examination was performed, and mandatory procedures such as enema, bladder catheterization, and blood transfusion preparation, if necessary, were carried out. Acetabular fractures were categorized using the Judet and Letournel classification system [8].

### Design and plate fabrication

**Figure 1:**
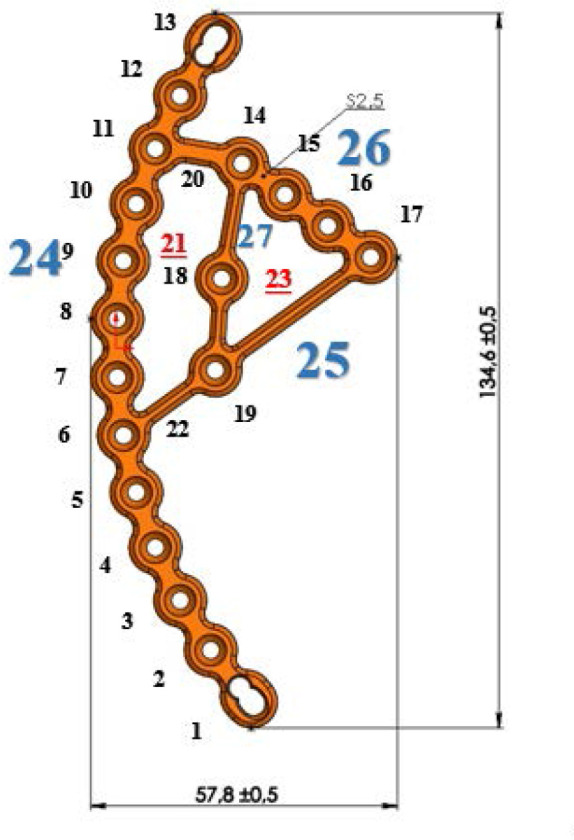
Schematic representation of the orthopedic plate

The developed pelvic plate, as shown in Figure 1 in the anterior projection, consists of the main part of the structure (24) bounded by two combined holes (1, 13) on the front and back, allowing for compression of the bone fragments and fixation. The inner part with a triangular hole (23) is separated from the superior crest portion (24) of the plate by two bridges (20, 22), creating an open space (21). Space 21 is surrounded by the main part (24), bridges 20 and 22, and the plate portion (27). Similar to other plates, this design includes a hole (18) for connection to an insertion instrument, which the surgeon will use during the surgery to implant the plate onto the pelvic bone. Hole 18 is a component of the plate portion (27). Plate portion 26 with holes and the plate portion 25 are connected in the area of the screw hole (17) on plate portion 26. Bridges 20 and 22 are relatively narrow in the model, allowing for easy bending of the inner part of the plate, which lies on the quadrilateral plate of the acetabulum, composed of plate portions 25, 26, and 27, in order to match the contour of the superior crest portion (24) of the plate.

The superior crest segment (24) of the plate has 13 screw holes. Two holes (1, 13) at the anterior and posterior edges are combined holes, while the remaining 11 holes have angular stability. These latter holes have internal threads on both the hole and the screw head, allowing for secure locking with 3.5 mm diameter screws (2–12). This provides additional rigidity to the construct. The part of the plate that contacts the quadrilateral plate is connected to the superior crest portion of the plate by bridge 22 in hole 6 and hole 19, while bridge 20 connects holes 11 and 14. In the plate portion (26) of the pelvic plate, there are 4 screw holes with angular stability (14-17) (Figure 1).

The plate is made of medical steel type 316L, characterized by high strength and, at the same time, ductility, which distinguishes it favorably from other types of metals. 316L steel is characterized by increased corrosion resistance due to the presence of metals such as chromium, nickel, and molybdenum. 316L steel is classified as paramagnetic and safe for MRI patients with these implants. However, this metal causes more artifacts compared to other metals. The material properties of this metal make it well-suited for orthopedic implants, addressing key requirements for surgeons. The metal structure is strong enough to withstand heavy loads after fixation to the bone, and its plasticity allows it to be easily contoured and fixed to the bone.

**Figure 2:**
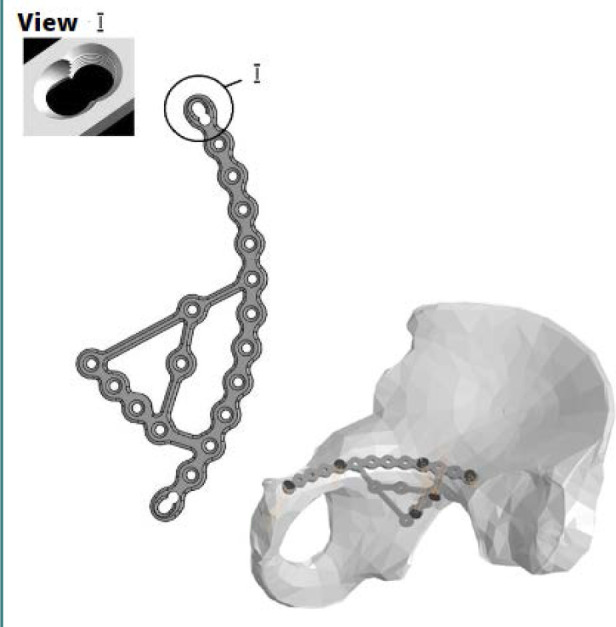
Orthopedic plate visualization (A) and pelvic system 3D model (B)

**Figure 3:**
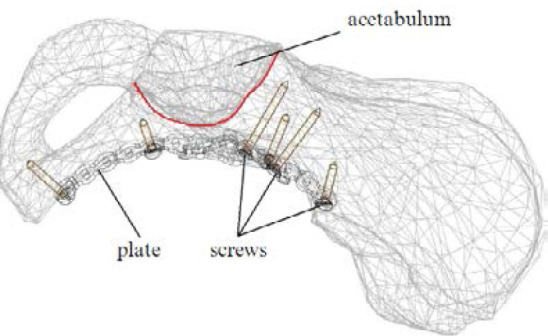
Safe insertion of screws into the pelvic bone

In the design of the pelvic plate, all holes feature angular stability, which ensures reliable fixation of both the bone fragments and the plate to the bone. The exception is the two outermost screw holes, which are designed as combined holes. This technical design allows for compression of the bone fragments and fixation, depending on the specific needs of the case. Additionally, the increased number of holes on both the quadrilateral plate side and the superior crest portion provides additional strength and versatility for the fixation of bone fragments, considering the nature of the fracture (Figures 2 and 3).

The complex anatomy and approach to the acetabulum cause significant technical problems in osteosynthesis, making it difficult to achieve ideal or optimal fixation of bone fragments using a plate. The developed plate model addresses these challenges by offering the bone varied and adaptable fixation options. The anatomical contouring and the increased number of holes allow the surgeon to achieve repositioning and fixation of bone fragments without serious technical difficulties. However, we acknowledge the potential for gaps in fixation and are committed to addressing these issues in future iterations of the plate.

**Figure 4:**
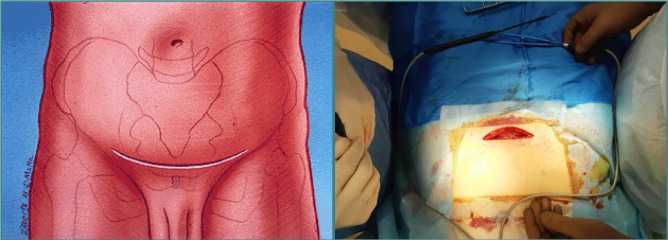
Modified Stoppa approach

The surgical procedure for utilizing this plate to fixate the acetabulum is performed as follows:

The patient is positioned supine on the operating table and administered endotracheal anesthesia. Using a modified Stoppa's technique for the anterior intrapelvic approach (Figure 4), an incision in the soft tissue is performed, located 2 cm above the pubic symphysis, transversely, with a length of 9.0 cm. The subcutaneous layer is mobilized, and the white line of the abdomen is identified and then longitudinally incised. The retroperitoneal space is exposed, and the urinary bladder is shifted posteriorly and downward. The symphysis pubis and pubic bones are identified along their superior, posterior, and anterior surfaces. The pubic-crest fascia is dissected and divided. The iliopectineal fascia is elevated, along with the vascular and nervous structures. An approach to the quadrilateral space is established, allowing for mobilization of the obturator nerve. Next, the terminal line and the fracture site are identified. Bone fragments are reduced using pelvic clamps and a piquer. The plate is contoured based on the nature and anatomy of the fracture by bending the quadrilateral and superior crest portions of the plate. The plate is adapted and positioned along the terminal line. The plate is fixed with screws starting from the iliosacral part towards the anterior portion of the pubic bone. X-ray control is performed, and a drain is placed in the operative area. The wound is meticulously closed in layers.

The assessment of reduction quality was determined according to Matta's criteria on pelvic X-rays. According to these criteria, a 0-1 mm displacement from the original position was classified as anatomical reduction, imperfect reduction as 2-3 mm displacement, and poor reduction as greater than 3 mm displacement [9]. Postoperative functional assessments of patients were carried out using the Harris Hip Score (HHS) scale [10,11].

### Statistical analysis

Statistical analysis was conducted using STATA statistical software. Descriptive statistics such as mean and standard deviation were used to describe quantitative variables, while frequency and percentage were utilized for qualitative variables.

## RESULTS

### Study population and fracture characteristics

Our study comprised eight participants, with a gender distribution of five men (62.5%) and three women (37.5%). The mean age was 41 years (SD = 10.5), with a median age of 40 and an interquartile range (IQR) of 33.5-48.5 years. While median surgery duration was similar to the mean value, median blood loss (375 ml) was significantly lower than average. This series included one anterior column fracture, two anterior column + posterior semi-transverse fractures, three both-column fractures, one T-shaped fracture, and one transverse fracture + posterior wall (Table 1). Among the patients included in this study, three injuries were due to falls from height, and five patients had injuries resulting from motor vehicle accidents. In all cases, the patients underwent surgical procedures utilizing the anterior intrapelvic approach, also known as the modified Stoppa approach, requiring additional superior pubic access in only one case. The patient and fracture characteristics are presented in Table 1.

**Table 1:** Patient characteristics, mechanism of injury, and fracture pattern

Parameter	Patients, *n* (%)
Cases	8
Sex Male Female	5 (62,5%) 3 (37,5%)
Mean ± SD age, y	41± 10,5
Injury mechanism Car accidents Fall	5 3
Fracture types Anterior column Anterior column + posterior hemi-transverse Transverse + posterior wall Both column T-type	1 (12,5%) 2 (25%) 1 (12,5%) 3 (37,5%) 1 (12,5%)

**Figure 5:**
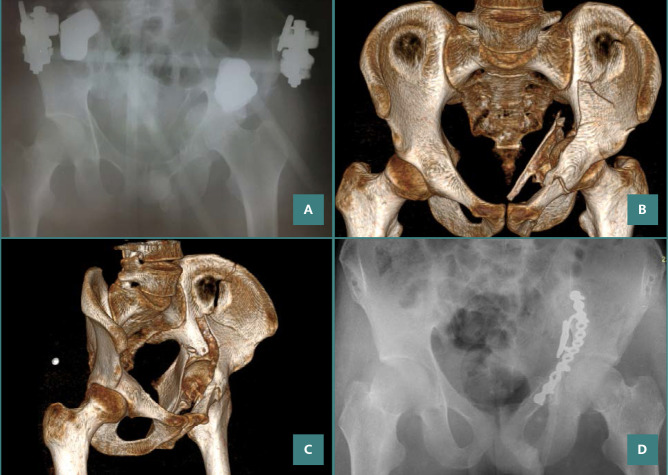
A 26-year-old patient with a both-column fracture of the acetabulum. Preoperative X-ray and CT sections: (A) preoperative direct X-ray, (B,C) preoperative CT sections, (D) X-ray on postoperative day 1.

**Figure 6:**
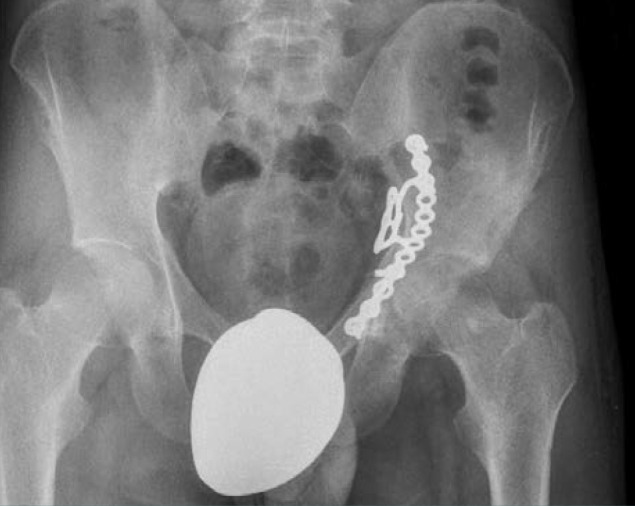
X-ray result after 6 months

The average duration of the surgery was 171.9 minutes (SD = 40.2). The mean intraoperative blood loss was 537.5 ml (SD = 521.5) (Table 2). Four patients required blood transfusion. When examining the X-rays based on Matta's criteria, the quality of the reduction was classified as anatomical in two cases (25%), imperfect in four cases (50%), and poor in two cases (25%) (Table 3, Figures 5 and 6).

**Table 2:** Mean surgery duration and blood loss

Variable	Total (*n* = 8)
Duration of operation in min, (mean ± SD)	171.9 ± 40.2
Blood loss in ml, (mean ± SD)	537.5 ± 521.5

**Figure 7:**
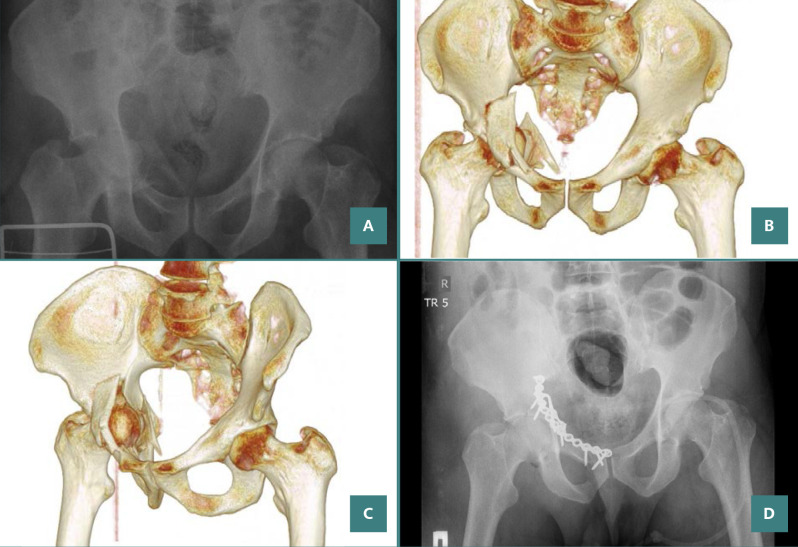
A 42-year-old patient with a both-column fracture of the acetabulum. Preoperative X-ray and CT sections: (A) preoperative direct X-ray, (B,C) preoperative CT sections, D) X-ray on postoperative day 1.

**Figure 8:**
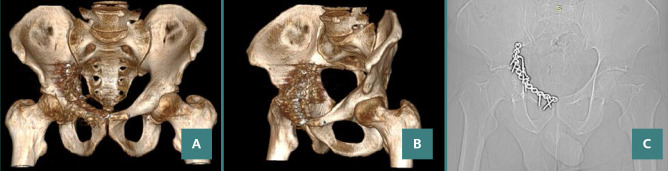
One-year postoperative imaging results with CT (A,B) and X-Ray (C)

**Figure 9:**
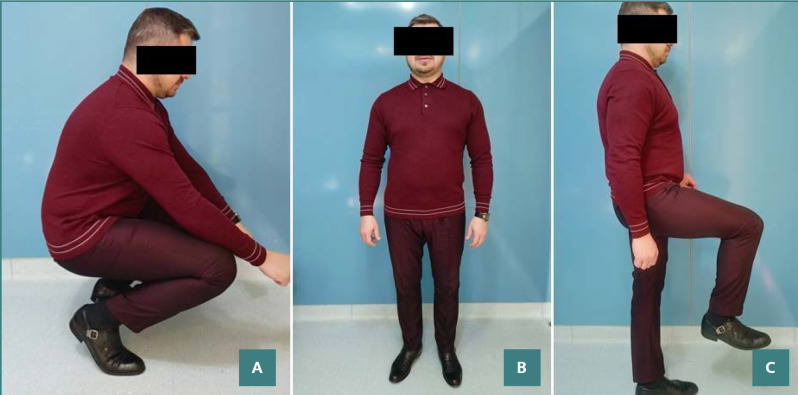
Photographs of the patient squatting (A), standing (B), and with the hip flexing (C) show that the involved hip joint had returned to normal function without limitation in range of motion and pain while walking.

One patient experienced an intraoperative injury to the superior gluteal vein, which was repaired during the surgery with the assistance of an angiosurgeon. Another patient had pre-existing post-traumatic neuropathy in the left lower limb because of the injury. During the one-year follow-up, one patient exhibited non-union of the fracture, the formation of a pseudoarthrosis of the acetabulum, and the development of post-traumatic arthrosis, which required subsequent surgery. Functional outcomes were assessed using the HHS approximately one year post-surgery. The results varied, with 37.5% of the cases (three patients) being classified as excellent, another 37.5% as good, 12.5% (one patient) as satisfactory, and the remaining 12.5% as poor, as detailed in Table 3. Another example of surgical treatment in a patient with an acetabular fracture is shown in Figures 7–9. Fractures of the acetabulum were encountered as isolated injuries in three patients, while five patients had associated injuries, including two head injuries, two upper limb injuries, and two lower limb injuries.

**Table 3:** Radiological outcomes and Harris Hip Scores distribution

	Total (*n* = 8)
Radiological outcome (Matta) Anatomical (<1 mm) Imperfect (2–3 mm) Poor (>3 mm)	2 (25%) 4 (50%) 2 (25%)
Harris Hip Score Excellent (90–100) Good (80–89) Fair (70–79) Poor (<70)	3 (37.5%) 3 (37.5%) 1 (12.5%) 1 (12.5%)

## DISCUSSION

An essential aspect of treating acetabular fractures is anatomic reduction and securing the bones firmly, which is vital for restoring joint alignment and minimizing the chances of osteoarthritis after injury. Letournel and Matta's research highlights that achieving precise alignment of fractures during surgery significantly impacts the ultimate clinical results [12]. Consequently, inadequate fracture reduction, characterized by displacement exceeding 3 mm, is now recognized as an unfavorable predictor of eventual functional recovery [12].

Using a quadrilateral plate for treating acetabular fractures presents a complex decision-making process. Multiple studies [1,13,14] indicate that conventional methods like plate or screw fixation might not offer sufficient stability for such fractures and can increase the likelihood of joint penetration, resulting in ineffective fixation and unfavorable results [15,16]. Hence, we developed a specific plate for stabilizing acetabular fractures. Our research findings validate that this innovative plate can lead to favorable clinical and radiographic results for fractures involving the quadrilateral plate. Using this plate for fixation in such cases could offer an excellent treatment alternative.

The conventional method for acetabular fracture repair typically involves the standard ilioinguinal approach pioneered by Letournel [8]. However, this approach carries risks of significant blood loss and morbidity, and mastering it demands extensive training due to the numerous critical anatomical structures involved. Alternatively, employing a new plate for managing quadrilateral plate fractures can be done using the Stoppa approach. This approach offers a more restricted exposure, potentially leading to shorter operative durations and reduced blood loss.

Compared to conventional plates, this novel design offers several potential benefits for acetabular fracture fixation. These include more secure fixation, improved anatomical contouring due to the plate's design, and enhanced strength and versatility for fragment fixation. These promising features warrant further investigation.

### Limitations

This study is limited by its small sample size and short follow-up period. Further evaluation of the efficacy of the plate in acetabular fractures requires larger, randomized, controlled trials with long-term follow-up to determine if this novel design offers significant advantages over conventional implants.

## CONCLUSION

The authors believe that the plate-assisted surgical treatment of acetabular fractures presented in this study is a promising option for the treatment of acetabular fractures. Early results using the plate with a modified Stoppa approach in a small patient cohort are encouraging, with good clinical outcomes (HHS) and radiographic reduction (Matta criteria). However, the limited sample size and short follow-up period necessitate further investigation. Future research should involve larger, randomized controlled trials with long-term follow-up to objectively evaluate the clinical efficacy of this plate in the surgical treatment of acetabular fractures. Currently, ongoing research aims to improve the management of acetabular fractures, addressing the modern requirements for managing high-energy trauma while overcoming the limitations of existing metal constructs used in acetabular fracture surgery. We believe that our development may be one of the options to solve the existing problems in the surgical treatment of acetabular fractures.
